# Predicting vision-threatening diabetic retinopathy in patients with type 2 diabetes mellitus: Systematic review, meta-analysis, and prospective validation study

**DOI:** 10.7189/jogh.14.04192

**Published:** 2024-10-11

**Authors:** Yanhua Liang, Xiayin Zhang, Wen Mei, Yongxiong Li, Zijing Du, Yaxin Wang, Yu Huang, Xiaomin Zeng, Chunran Lai, Shan Wang, Ying Fang, Feng Zhang, Siwen Zang, Wei Sun, Honghua Yu, Yijun Hu

**Affiliations:** 1 Guangdong Eye Institute, Department of Ophthalmology, Guangdong Provincial People’s Hospital (Guangdong Academy of Medical Sciences), Southern Medical University, Guangzhou, China; 2 Department of Ophthalmology, The People’s Hospital of Jiangmen, Southern Medical University, Jiangmen, China; 3 Department of Endocrinology, Nanhai District People’s Hospital of Foshan, Foshan, China; 4 Guangdong Provincial Key Laboratory of Artificial Intelligence in Medical Image Analysis and Application, Guangzhou, China

## Abstract

**Background:**

Delayed diagnosis and treatment of vision-threatening diabetic retinopathy (VTDR) is a common cause of visual impairment in individuals with type 2 diabetes mellitus (T2DM). Identification of VTDR predictors is the key to early prevention and intervention, but the predictors from previous studies are inconsistent. This study aims to conduct a systematic review and meta-analysis of the existing evidence for VTDR predictors, then to develop a risk prediction model after quantitatively summarising the predictors across studies, and finally to validate the model with two Chinese cohorts.

**Methods:**

We systematically retrieved cohort studies that reported predictors of VTDR in T2DM patients from PubMed, Ovid, Embase, Scopus, Cochrane Library, Web of Science, and ProQuest from their inception to December 2023. We extracted predictors reported in two or more studies and combined their corresponding relative risk (RRs) using meta-analysis to obtain pooled RRs. We only selected predictors with statistically significant pooled RRs to develop the prediction model. We also prospectively collected two Chinese cohorts of T2DM patients as the validation set and assessed the discrimination and calibration performance of the prediction model by the time-dependent ROC curve and calibration curve.

**Results:**

Twenty-one cohort studies involving 622 490 patients with T2DM and 57 107 patients with VTDR were included in the meta-analysis. Age of diabetes onset, duration of diabetes, glycosylated haemoglobin (HbA1c), estimated glomerular filtration rate (eGFR), hypertension, high albuminuria and diabetic treatment were used to construct the prediction model. We validated the model externally in a prospective multicentre cohort of 555 patients with a median follow-up of 52 months (interquartile range = 39–77). The area under the curve (AUC) of the prediction model was all above 0.8 for 3- to 10-year follow-up periods and different cut-off value of each year provided the optimal balance between sensitivity and specificity. The data points of the calibration curves for each year closely surround the corresponding dashed line.

**Conclusions:**

The risk prediction model of VTDR has high discrimination and calibration performance based on validation cohorts. Given its demonstrated effectiveness, there is significant potential to expand the utilisation of this model within clinical settings to enhance the detection and management of individuals at high risk of VTDR.

The International Diabetes Federation has estimated that the global prevalence of diabetes in individuals aged 20–79 years was 10.5% (536.6 million people) in 2021, with a projected increase to 12.2% (783.2 million people) by 2045 [1]. Among the various microvascular complications of diabetes, diabetic retinopathy (DR) is prevalent and it can develop into vision-threatening diabetic retinopathy (VTDR) without exhibiting any symptoms [2]. Vision-threatening diabetic retinopathy is characterised by the presence of severe non-proliferative DR, proliferative DR, and/or macular edema. Diabetic retinopathy and diabetic macular edema (DME) are diagnosed and graded according to the International Clinical Severity Scale of Diabetic Retinopathy and DME (Figure S1 in the **Online Supplementary Document**). A meta-analysis has reported a global prevalence of VTDR of 6.17% in patients with diabetes [3]. Therefore, regular fundus examinations in diabetic patients are crucial for early detection and timely treatment of VTDR.

However, providing regular DR screening on a global scale poses significant challenges. The need for frequent screening incurs high financial costs and many developing countries and economically disadvantaged areas are short of the necessary equipment and physicians for conducting fundus screening. To identify populations 'at risk' of developing VTDR, researchers in recent years have explored early predictive markers for VTDR, such as duration of diabetes, glycosylated haemoglobin (HbA1c), albuminuria, and diabetes medication [4–6]. Nevertheless, there are controversies surrounding the roles of certain predictors, including sex, body mass index (BMI), total cholesterol, and statin use [7–9]. Despite these individual studies, a comprehensive and systematic review assessing the predictors of VTDR is warranted.

Prior research has examined the predictors of DR [10,11], yet comprehensive assessment and meta-analysis of the predictors of VTDR are lacking. Furthermore, while predictive models for DR or DME have been developed in previous studies [12,13], the predictive model for VTDR has not been established until 2022, and they are flawed with short follow-up duration [2], being cross-sectional studies or case-control studies, low accuracy [14,15], lack of external validation [16] and incorporating complex system variables that were not easily accessible [17]. Therefore, we conducted a comprehensive systematic review and meta-analysis to identify independent predictors of VTDR, and then developed a risk prediction model based on these independent predictors and validated it using data collected from two Chinese cohorts.

## METHODS

In the present study, we first conducted a systematic review and meta-analysis to summarise the predictors of VTDR, followed by the development of a VTDR prediction model using a scoring system based on these predictors. The discrimination and calibration performance of the prediction model was further validated by two Chinese cohorts using the time-dependent receiver operating characteristic (ROC) curve and calibration curve.

### Study registration

This study was conducted following the Preferred Reporting Items for Systematic Review and Meta-analysis (PRIMA) guidelines [18] and it was registered on PROSPERO with unique identifier: CRD42022380342. The detailed registration document is shown on the website (https://www.crd.york.ac.uk/prospero/).

### Eligibility criteria

The inclusion criteria were as follows:

(1) Patients aged over 18 years old and diagnosed with T2DM;

(2) T2DM and VTDR were medically confirmed by clear and reasonable diagnostic criteria;

(3) Predictors of VTDR were analysed using either multivariable logistic regression or multivariable cox regression;

(4) The original studies were cohort studies investigating predictors of VTDR.

The exclusion criteria were as follows:

(1) Patients diagnosed with T1DM, gestational diabetes, or other specific types of diabetes, such as diabetes secondary to malignancies or other metabolic disorders;

(2) Patients with comorbidities, including severe systemic disease or eye disease including glaucoma, age-related macular degeneration, other retinal vascular diseases, and refractive interstitial opacification such as severe cataracts;

(3) Studies that did not control for other confounding factors or did not perform multivariable regression analysis;

(4) Cohort studies that were published in full text without undergoing a peer review process.

### Literature search

A comprehensive search was conducted in seven English databases to collect relevant studies, including PubMed, Ovid, Embase, Scopus, Cochrane Library, Web of Science, and ProQuest, from inception to December 2023. The search strategy comprised a combination of text and Medical Subject Headings (MeSH) related to ‘Diabetic Retinopathy’, ‘vision-threatening’ and ‘risk factor’. The detailed search strategy is presented in Table S1 in the **Oline Supplementary Document**).

### Study selection

All the retrieved articles were imported into EndNote (version X9, Clarivate Analytics). Duplicated articles, reviews, meta-analyses, animal experiments, non-English studies, conference abstracts, case reports, guidelines, letters, editorials, book chapters, and clinical trial protocols were removed. Irrelevant studies were excluded according to the title and abstract. The remaining articles were downloaded, read comprehensively, and carefully selected based on the inclusion and exclusion criteria.

### Data extraction

The following information was extracted and entered into a database: study design, location and published year, patient demographic characteristics (age and sex), numbers of patients enrolled and onset numbers, fundus examination at baseline, duration of the follow-up, identified risk factors and their RRs with corresponding 95% CIs. To minimise heterogeneity, for duplicates, we only included the one with the most comprehensive information. Additionally, we recorded the RR values and 95% CIs of the predictors obtained from multivariate regression analyses.

### Quality assessment

The quality of cohort and case-control studies was assessed by using the Newcastle–Ottawa scales (NOS). The NOS contains eight items that are categorised into three dimensions: selection, comparability, and outcome. A star system is used to allow a semiquantitative assessment of study quality. The maximum number of stars awarded per item is one, except for the item related to comparability that allows the assignment of two stars. The NOS ranges between zero and nine stars [19]. Studies with scores of 7 or higher are considered high quality, 4 to 6 are considered medium quality, and 3 or below are considered low quality.

Two investigators (L-YH and Z-XY) independently conducted literature screening, study selection, data extraction, and quality assessment. Any discrepancies after cross-checking were resolved by consulting two other senior investigators (Y-HH and H-YJ) to reach a consensus.

### Validation cohorts

Two validation cohorts were collected from People’s Hospital of Jiangmen, Southern Medical University and Nanhai District People’s Hospital of Foshan. The study was approved by the Ethics Committee of both hospitals (The ethical approval numbers for People’s Hospital of Jiangmen and Nanhai District People’s Hospital are 20220302-8 and 2023266 respectively). The validation cohort data collected at Jiangmen People's Hospital were from the inpatient electronic medical record system of the T2DM population hospitalised in the Department of Endocrinology from 2015 to 2019. The validation cohort data collected at Nanhai District People's Hospital was from the diabetes electronic medical record system of the T2DM population who were seen at the Metabolic Management Centre (MMC) since 2018. Each patient in the electronic medical record system had a unique identification number.

The inclusion criteria for the validation cohort were as follows:

(1) patients older than 18 years old and diagnosed with type 2 diabetes;

(2) Patients were hospitalised or visited the clinic at least twice, with a follow-up for more than 36 months;

(3) Patients had fundus examinations at both baseline and last follow-up, and not diagnosed with VTDR at baseline. Patients with glaucoma, age-related macular degeneration, and other retinal vascular diseases were excluded.

#### Evaluation and diagnosis of VTDR

The cohort population underwent a standardised clinical examination and retinal photography. DR was graded by trained and certified ophthalmologists according to the American Academy of Ophthalmology (AAO) International Clinical Diabetic Retinopathy Disease Severity Scale at both medical centres. DR was defined as the presence of non-proliferative DR (NPDR), proliferative DR (PDR), diabetic macular oedema (DME), or any combinations thereof, meanwhile vision-threatening DR (VTDR) was defined as the presence of severe NPDR, PDR, and/or DME [20]. Patients were excluded if fundus photographs could not be graded due to missing data or nondiagnostic images due to cataracts or vitreous clouding. If a photograph of one eye could not be identified, the final diagnosis was determined based on the only photograph available.

#### Collection of basic information and blood biochemical data

Basic clinical information included the patient's unique identification number, gender, age, and type of diabetes (as diagnosed by the medical centre's endocrinologist). Age of diabetes onset, duration of diabetes mellitus, history of insulin therapy, and history of hypoglycaemic medication were obtained from the electronic medical record system records. Fasting (eight hours) venous blood samples were collected for analysis of serum creatinine (Scr), HbA1c, total cholesterol (TC), high-density lipoprotein cholesterol (HDL-c), low-density lipoprotein cholesterol (LDL-c), triglycerides (TG), and serum uric acid according to the standardised procedures of certified laboratories in China. Urine samples were also obtained for analysis of urinary albumin and urinary creatinine. Estimated glomerular filtration rate (eGFR) (male) = [(140–age) × body weight (Kg)] / [0.818 × blood creatinine (μmol/L)] (eGFR (women) = eGFR (male) × 0.85). High albuminuria was defined as urinary albumin-to-creatinine ratio (UACR)>30 mg/g.

### Statistical analysis

#### Meta-analysis

The predictors involving more than two cohort studies were included in the meta-analysis. RRs with 95% CIs for each predictor of VTDR were extracted and then the pooled RRs were generated using a fixed effects model or a random effects model according to the heterogeneity. Heterogeneity across studies was assessed using the Cochrane Q test and measured by *I^2^*. An *I^2^*>50% indicated high heterogeneity, and a sensitivity analysis was required, which were conducted by omitting a single study in turn to test the robustness of the results. If omitting a single study resulted in an *I^2^*<50%, a fixed effects model was used, otherwise a random effects model was employed. If a predictor included both continuous and categorical variables, subgroup analyses were performed depending on the type of variable. All tests were two-sided and statistical significance was defined as *P* < 0.05. All analyses were performed using Stata software version 15.0 (StataCorp, College Station, TX).

#### Model development

We used a categorisation point system, as suggested by Sullivan et al. [21], to develop our prediction model. First, all predictors included in the prediction model were selected from earlier analyses. Second, the pooled RR of each predictor was extracted. Subgroup analysis would be performed if a predictor had both categorical and continuous variable forms. If the categorical variables had the same truncation value and there were more studies available, we prioritised the pooled RR of the subgroup containing categorical variables for model development, otherwise, we selected the subgroup with continuous variables. The β-coefficient of each predictor was calculated according to the pooled RR and its corresponding 95% CI. Then, scores were derived by multiplying the β-coefficients by 10 and rounding to one decimal place. Finally, all predictors in the model were categorised based on the meta-analysis and clinical practice guidelines [22–24], and each category was assigned a score. Statistical significance was determined at a *P*-value <0.05.

#### External multicentre validation

The external cohort data described above were used to examine the discrimination and calibration of the prediction model and identify the best implementation strategy. The total score for each individual in the validation cohort was calculated by summing the scores of all components in the risk prediction model developed above. Then the total score, duration of follow-up and whether VTDR occurred at the endpoint of follow-up for each individual in the validation cohort were used to make time-dependent ROC curve and calibration curve. Sensitivity, specificity and area under the curve (AUC) were calculated at different follow-up time points and cut-off values. The results were used to determine the optimal cut-off point [25] at different follow-up times. All analyses were performed using MedCalc Statistical Software (Version 18.2.1; MedCalc Software bvba, Ostend, Belgium; http://www.medcalc.org; 2018) and R version 4.2.1 (R Foundation for Statistical Computing, Vienna, Austria).

## RESULTS

### Study selection

**Figure 1:**
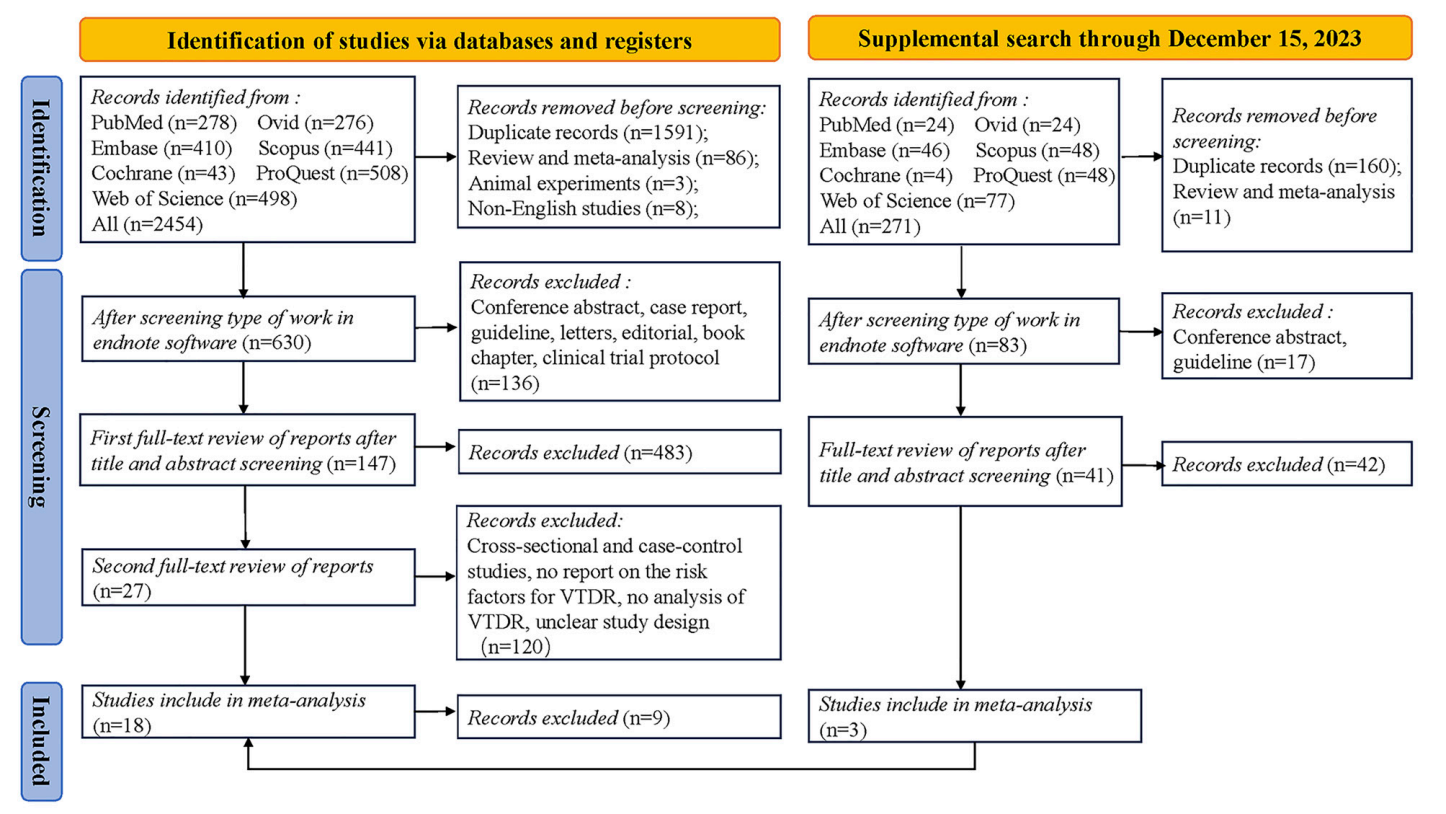
Flowchart outlining the literature search and study selection for predictors of vision-threatening diabetic retinopathy (VTDR) development in patients with type 2 diabetes mellitus (T2DM).

Initially, we successfully collected 2454 articles and finally 18 articles met the inclusion criteria after identification and screening. We conducted a supplemental literature search in December 2023 and collected a total of 271 articles, which were screened based on inclusion and exclusion criteria and obtained 3 articles. The study selection methodology is shown in **Figure 1**.

### Characteristics and quality of included studies

Among the 21 included studies, 13 studies were prospective cohorts and 6 studies were retrospective cohorts. These studies collectively involved a total of 622 490 patients with T2DM, of which 57 107 VTDR cases were observed during follow-up. The duration of follow-up ranged from three to 10 years, with a maximum follow-up period of 17 years. The average age of the study participants was between 53 and 68 years old and the duration of diabetes varied from one to 12 years. The cohorts were from Europe (England and Spain), Asia (China, Iran, and India), and the USA. About 78% of the participants (n = 488 413 participants) were from Europe and the USA, while the remaining 22% (n = 134 077 participants) were from Asia. The characteristics of all these 21 cohorts are presented in Table S2 in the **Online Supplementary Document**. According to the NOS, all of the included studies had a score of seven or higher, which indicated high quality study. Detailed information on NOS is shown in Table S3 in the **Online Supplementary Document**.

### Meta-analysis

There were 14 predictors identified in two or more articles, including age, race, sex, BMI, diabetes duration, HbA1c, hypertension, total cholesterol, eGFR, high albuminuria, waist-to hip ratio (WHR), statin use, anti-hypertensive medication, and diabetes treatment. The factor ‘age’ refers to the age of diabetes onset in some articles and the age at baseline in others. To ensure the independence of the study variables, we chose to focus on the ‘age of diabetes onset’ rather than ‘age at baseline’ as the selected factor. Detailed information on these predictors is shown in Table S4 in the **Online Supplementary Document**.

Of the 14 predictors included in meta-analysis, eight factors were associated with VTDR development, including the age of diabetic onset, diabetes duration, HbA1c, hypertension, BMI, eGFR, high albuminuria and diabetic treatment. The factors 'duration of diabetes' and 'HbA1c' included both continuous and categorical variables, therefore subgroup analyses were conducted according to the type of variable. There were different definitions of ‘overweight’ and ‘obese’ among studies in different countries or regions involving the factor ‘BMI’ which contributed to greater heterogeneity. Thus, ‘BMI’ was not included in the final risk prediction model. Detailed information on pooled RR is shown in Table S5 in the **Online Supplementary Document****.**

### Model derivation

Seven predictors associated with VTDR were used to construct the prediction model, including the age of diabetes onset (<50 years, RR = 1.044; 95% CI = 1.005–1.085), diabetes duration (per one year increase, RR = 1.083; 95% CI = 1.063–1.104), HbA1c (per 1% increase from 7%, RR = 1.171; 95% CI = 1.041–1.317), hypertension (RR = 1.919; 95% CI = 1.789–2.059), eGFR (<60 ml / min / 1.73m^2^, RR = 1.358; 95% CI = 1.226–1.505), high albuminuria (RR = 1.585; 95% CI = 1.265–1.985), diabetic treatment (oral drugs, RR = 1.63; 95% CI = 1.08–2.47); insulin, RR = 2.88; 95% CI = 1.47–5.64)). Predictors with the number of involved studies, sample size, pooled RRs (95%CI), β-coefficients, and risk scores included in the VTDR risk prediction model can be found in Table S6 in the **Online Supplementary Document**.

Conclusively, a simple risk-scoring tool for VTDR was established as follows: age of diabetes onset (years; ≥50, scores 0; <50, scores 0.4), duration of diabetes (scores 0.8 per one year), HbA1c (≤7%, scores 0; per 1% increment, scores 1.6), hypertension (non-hypertension, scores 0; hypertension, scores 6.5), eGFR (ml/min/1.73m^2^, ≥60, scores 0; <60, scores 3.1), high albuminuria (non-high albuminuria, scores 0; high albuminuria, scores 4.6) and diabetic treatment (no treatment, scores 0; oral drugs, scores 4.9; insulin treatment, scores 10.6). The detailed predictors and their corresponding scores are shown in **Table 1**.

**Table 1:** Vision-threatening diabetic retinopathy (VTDR) risk prediction model for patients with type 2 diabetes mellitus (T2DM)

Predictors of VTDR	Category	Points
Age of diabetes onset (years)	≥50	0.0
	<50	0.4
Duration of diabetes (years)	0	0.0
	Per 1 y increase	0.8
HbA1c (1%)	≤7	0.0
	Per 1% increase	1.6
Hypertension*	No	0.0
	Yes	6.5
eGFR (ml/min/1.73m^2^)	≥60	0.0
	<60	3.1
High albuminuria†	No	0.0
	Yes	4.6
Diabetic treatment	No	0.0
	Oral drugs	4.9
	Insulin	10.6

HbA1c – glycosylated haemoglobin, eGFR – estimated glomerular filtration rate, VTDR – vision-threatening diabetic retinopathy

*Hypertension was defined as systolic blood pressure greater than 140 mm Hg.

†High albuminuria was defined as urinary albumin-to-creatinine ratio (UACR)>30mg/g.

### Baseline characteristics of two validation cohorts

The validation cohorts from two Chinese tertiary care hospitals included a total of 555 patients with T2DM (291 in cohort 1 and 264 in cohort 2). Of these, 263 patients (47.4%) were male, and 50 patients (9.9%) developed VTDR during the follow-up period (52 months, IQR = 39–77). The survival curve of the VTDR endpoint of the validation cohort is shown in Figure S2 in the **Online supplementary Document**. The mean age of diabetes onset at baseline was 50.8 ± 11.5 and the median duration of diabetes was 71.5 months (IQR = 22–123). Compared to those who did not develop VTDR, patients who developed VTDR had a longer duration of diabetes at baseline (63.5 months, IQR = 18.3–120 vs. 114 months, IQR = 48–150), higher HbA1c (7.4%, IQR = 6.5–9.5 vs. 10.7%, IQR = 8.1–11.9), greater proportion of patients with hypertension (46.9 vs. 64.0%), eGFR<60 ml / min / 1.73 m^2^ (15.8 vs. 28.0%), albuminuria (16.6 vs. 54.0%), insulin use (39.6 vs. 90.0%), but a smaller proportion of patients taking oral diabetic medications (64.0 vs. 46.0%). The baseline characteristics of patients in the validation cohorts are presented in Table S7 in the **Online Supplementary Document**.

### Model validation

**Figure 2:**
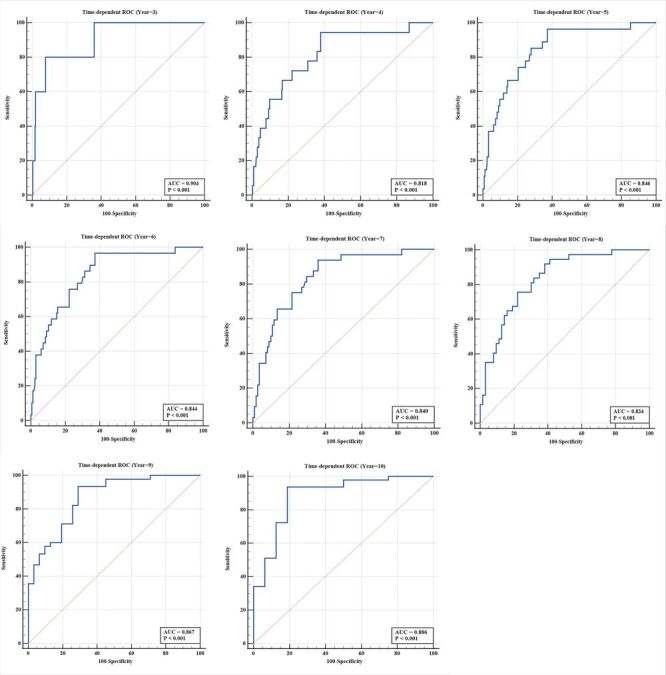
Time-dependent receiver operating characteristic (ROC) curve of the vision-threatening diabetic retinopathy (VTDR) prediction model for different years (year = 3, 4, 5, 6, 7, 8, 9, 10). The area under the curve (AUC) of the prediction model was all above 0.8 for 3- to 10-year follow-up periods.

**Figure 3:**
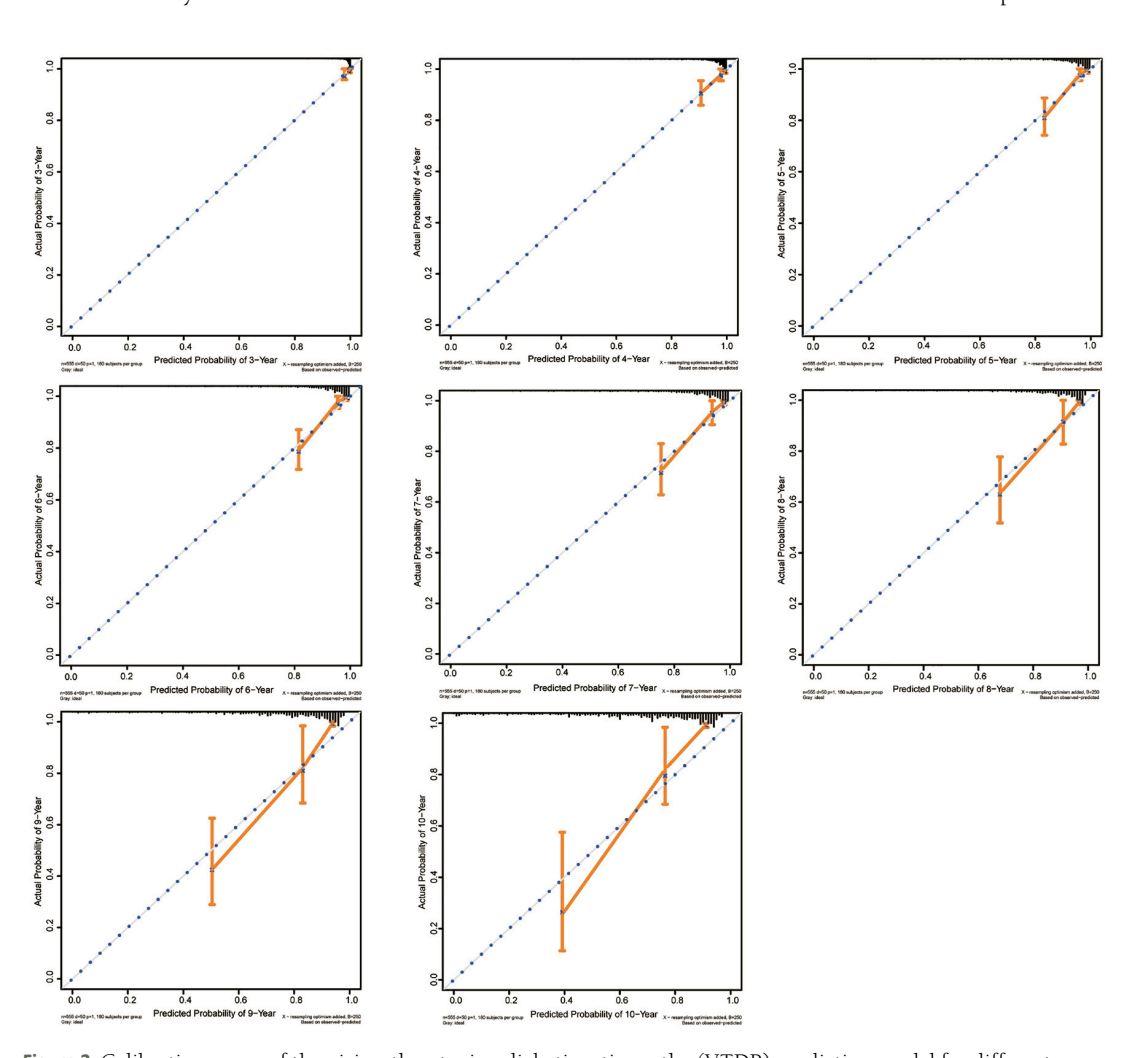
Calibration curve of the vision-threatening diabetic retinopathy (VTDR) prediction model for different years (year = 3, 4, 5, 6, 7, 8, 9, 10). The data points of the calibration curves for each year closely surround the corresponding dashed line and largely overlap the dashed line overall, and furthermore, the slopes of the calibration curves for each year are close to one.

The time-dependent ROC curve and calibration curve (year = 3, 4, 5, 6, 7, 8, 9, 10) of the VTDR prediction model is shown in **Figure 2** and the corresponding AUC, optimal cut-off values, and sensitivity/specificity are presented in **Table 2**. Detailed sensitivity and specificity at different follow-up times and cut-off values are shown in Table S8 in the **Online Supplementary Document**. Given that the median follow-up time for the validation cohorts was 52 months (IQR = 39–77), our prediction model focused on predicting VTDR within four and five years using the optimal cut-off value. The AUC values were 0.818 (95% CI = 0.773–0.857), 0.846 (95%CI = 0.797–0.887) for the 4th and 5th follow-up years, respectively. Moreover, a risk cut-off of 23.0 was selected as the optimal for the 4th and 5th follow-up years, resulting in a sensitivity and specificity of 94.44 / 61.98%, 96.30 / 62.70% respectively. The calibration curve showed our prediction model had good performance which is shown in **Figure 3**. The data points on the curve were fairly close to the ideal perfectly fitted line, and the slope of the calibration curve was close to one over the entire risk range.

**Table 2:** Performance and cut-off of vision-threatening diabetic retinopathy (VTDR) risk prediction model at different follow-up times

Follow-up, time (year)	Cut-off value*	Sensitivity/specificity, %*	Youden index	AUC*	95% CI (AUC)
3	23.8	100.00 / 64.00	0.640	0.904	0.877 ~ 0.928
4	23.0	94.44 / 61.98	0.564	0.818	0.773 ~ 0.857
5	23.0	96.30 / 62.70	0.590	0.846	0.797 ~ 0.887
6	23.0	96.55 / 62.65	0.592	0.844	0.786 ~ 0.892
7	22.7	93.75 / 63.96	0.572	0.840	0.769 ~ 0.896
8	22.7	91.89 / 61.90	0.538	0.834	0.746 ~ 0.901
9	21.9	93.33 / 70.97	0.643	0.867	0.769 ~ 0.934
10	21.9	93.62 / 81.25	0.749	0.886	0.780 ~ 0.952

AUC – area under the curve, CI – confidence interval

*The AUC values were 0.904, 0.818, 0.846, 0.844, 0.840, 0.834, 0.867, 0.886 respectively for 3th, 4th, 5th, 6th, 7th, 8th, 9th, and 10th follow-up years, respectively. Since the model was developed for an early detection of high-risk VTDR, a risk cut-off of 23.8, 23.0, 23.0, 23.0, 22.7, 22.7, 21.9,21.9 was selected as the optimal risk cut-off respectively for the 3th, 4th, 5th, 6th, 7th, 8th, 9th, and 10th follow-up years, resulting in a sensitivity and specificity of 100.00 / 64.00%, 94.44 / 61.98%, 96.30 / 62.70%, 96.55 / 62.65%, 93.75 / 63.96%, 91.89 / 61.90%, 93.33 / 70.97%, 93.62 / 81.25%.

## DISCUSSION

The progression of DR follows a particular pattern that takes several years from non-proliferative DR (NPDR) to VTDR [26]. A meta-analysis reported that among people with T2DM who had either no or mild DR at baseline, the incidence of VTDR was approximately one per 100 person-years and eight per 100 person-years, respectively [27]. Based on retinal screening at the patient’s last follow-up visit, several studies recommended extending the screening interval to two or three years for T2DM patients without DR [28–32]. A study demonstrated that a three-year screening interval for people with diabetes was most likely to be cost-effective [33]. However, we believe that developing VTDR screening strategies based on individual risk profiles is the most cost-effective approach for society.

Recently, different types of studies, including cross-sectional studies, case-control studies, and cohort studies, aim to explore multiple risk factors associated with VTDR. However, the findings are not completely consistent across these studies [2,34,35]. Therefore, we opted to employ a systematic review and meta-analysis to integrate cohort studies and specifically focused on the development of VTDR. Through this approach, we aimed to identify and summarise risk factors associated with incident VTDR and construct a simple clinical prediction model with prediction accuracy. The developed model is expected to identify populations at high risk of developing VTDR, hence providing an ‘early warning’ of VTDR and reducing the financial burden on the patients. Moreover, it allows for the efficient allocation of social health care resources, especially in resource-constrained areas where systematic retinal screening is not readily available.

In our systematic review and meta-analysis, 21 cohort studies were included and seven independent risk factors were screened out, including age of diabetic onset, duration of diabetes, HbA1c, hypertension, eGFR, high albuminuria and antidiabetic therapies (insulin and oral drugs). Regarding age, several studies have included ‘age at baseline’ in their statistical analyses, but the results have been inconsistent. Some studies showed that the risk of VTDR was not correlated with the baseline age [9,36], while others showed a decrease in VTDR risk with increasing baseline age [8,37]. Conversely, a study indicated that either a younger (<40 years) or older (>70 years) baseline age increased the risk of VTDR [4]. Considering that baseline age is the sum of the age of diabetic onset and duration of diabetes, our study demonstrated that the risk for VTDR increased by 8.3% per year. Therefore, the definition of age was carefully distinguished in our meta-analysis, and the age of diabetic onset was selected for statistical analysis. Our study revealed that the age of diabetic onset <50 increased the risk of VTDR by 4.4%. Furthermore, insulin treatment emerged as the most powerful risk factor, with a 1.88-fold increased risk of VTDR. In addition, an increase in HbA1c level by 1% corresponded to a 17.1% increase in VTDR risk, and hypertension nearly doubled the risk of VTDR. Hyperglycaemia and hypertension have long been shown to be independent risk factors for DR. A previous report suggested that interventions to lower blood pressure had a beneficial effect in preventing DR, particularly in hypertensive T2DM patients [38]. The ACCORD Eye study, which included 2856 T2DM patients at high risk for cardiovascular disease, demonstrated that intensive blood glucose control therapy (target HbA1c <6.0%) significantly reduced the rate of DR progression by 23% [39]. A study showed that a decrease in eGFR (<60 mL / min / 1.73 m^2^) and the presence of albuminuria (≥30 μg/mg) at baseline significantly increased the risk of VTDR during follow-up [6]. The relationship between chronic kidney disease (CKD) and DR has been investigated in several cross-sectional studies, with albuminuria identified as a reliable marker of D [40,41]. For instance, a cross-sectional study involving over 28 000 patients in Spain reported that eGFR levels <45 ml / min / 1.73 m^2^ were associated with a higher prevalence of DR. Moreover, individuals with elevated UACR had a higher prevalence of DR than those with reduced eGFR [42]. Additionally, our meta-analysis also showed similar results where higher UACR was associated with a higher risk of VTDR than lower eGFR. Specifically, patients with UACR>30 mg/g showed 58.5% increased risk of VTDR, while eGFR<60 ml / min / 1.73 m^2^ corresponded to a 35.8% increased risk of VTDR.

Regarding BMI, the study showed that both overweight and obesity were associated with a reduced risk of VTDR. In particular, the risk of VTDR in overweight and obese patients was 71.3 and 55.1% respectively compared to normal-weight patients. In recent years, studies conducted in Asian populations had similar findings. The Singapore Eye Disease Epidemiology Cohort Study found that BMI levels were negatively associated with any DR and VTDR [43]. The Shanghai Diabetes Registry study, which included 2533 T2DM patients, also reported that overweight patients had a lower risk of DR than normal-weight patients [44]. However, it is important to note that some studies showed no association between BMI and the risk of DR [45–47], while others demonstrated an increased risk of DR with a higher BMI[48, 49]. There were some reasons for excluding BMI in our prediction model. First, there were different definitions of ‘overweight’ and ‘obese’ among studies involving the factor ‘BMI’ which may introduce greater heterogeneity. Second, there have been conflicting results from epidemiological studies exploring the relationship between DR and BMI.

This study developed and validated a prediction model to help physicians identify patients at high risk of VTDR who should be referred to an ophthalmologist. The prediction model was transformed into a straightforward risk score, so that the technique could be applied in clinical practice to help people better control the risk components in the model. Endocrinologists, community physicians, primary care physicians, and other non-ophthalmic physicians could easily stratify VTDR risk in T2DM patients based on the prediction model or risk score. In an ongoing project, we aim to integrate this model with the Clinical Decision Support System (CDSS) to enable it to automatically collect data on seven system characteristics and alert doctors to patients who may be at high risk of VTDR. Besides physicians, in the future T2DM patients may even utilise the VTDR risk scores for self-assessment and risk management, since it is developed using easily accessible parameters.

This study is the first review and meta-analysis to comprehensively evaluate the VTDR predictors. Our innovation lies in constructing the model based on the combined RR values of independent predictors obtained from previous reviews and meta-analyses. The validation of the VTDR model makes up for the limits of previous studies. First, our study included a validation set of T2DM patients from two medical centres with a median follow-up of 52 (IQR = 39–77) months. The longer follow-up duration of validation cohorts allows our prediction model to estimate the risk of VTDR for three to 10 years. Second, time-dependent ROC curve analysis for three to 10 years yielded an AUC value above 0.8, suggesting good discrimination performance. The cut-off values at different follow-up time points were selected to improve the sensitivity and specificity of the model. This model can dynamically assess VTDR risk in T2DM patients to allow for in-time adjustment of their treatment regimens accordingly. Third, the predictive performance of our model was thoroughly evaluated via the calibration curves, which indicated a close-to-ideal slope of 1, suggesting good calibration performance. In addition, the model’s utilisation of easily accessible system variables and avoidance of complex calculations, make it convenient and intuitive for both patients and clinicians. Early prediction and appropriate intervention may hopefully help individuals reduce the risk of VTDR. Nevertheless, there are several limitations to our study. First, the method of systematic review and meta-analysis was inevitably heterogeneous due to variations in research designs and methods. We did not include duplicate literature data, extracted only the results of multivariate analyses during data extraction, and performed subgroup analyses and sensitivity analyses during data analyses, thus reducing the heterogeneity. However, despite these efforts, uncontrollable heterogeneity persisted due to incomplete consistency of the variables analysed in the multivariate analyses in each study, and inconsistency in the follow-up duration across studies. Second, our study involved 78% European and American patients and 22% Asian patients, which may affect the generalisability of our prediction model. Additional cohort studies from different ethnic groups or countries are required to generate a robust and reliable VTDR predictive model for general population. In addition, although the model uses easily accessible systematic variables and does not require complex calculations, it is imperative to gather more patient data from different health care organisations or clinics to validate and optimise the model, thereby further improving its applicability in different clinical settings. Third, in the meta-analysis, only the predictors involving more than two studies were included, which may have overlooked some predictors that were less reported but may have better predictive performance during modelling. Finally, we constructed an incidence prediction model with a 3- to 10-year follow-up period, which is a longer prediction time than previous VTDR models. Given the median follow-up periods was 52 months, the number of patients with relatively long follow-up periods was small, which may affect the long-term performance and reliability of the model to some extent. In the future, we plan to include more patients with longer follow-up periods for model validation, thus increasing the utility and robustness of the model.

## CONCLUSIONS

In conclusion, we have developed a simple and robust risk prediction model for VTDR at different follow-up time points, based on medical history and clinical laboratory parameters, including age of diabetic onset, duration of diabetes, HbA1c, hypertension, eGFR, albuminuria, and diabetes treatment. Subsequently, the model was validated using external cohorts, which confirmed its good discrimination and calibration performance for incident VTDR in T2DM patients. Moreover, the validated model provides a valuable tool for early prevention and personalised intervention strategies targeting VTDR.

## Additional material


Online Supplementary Document
