# Supplementary material for: Predicting vision-threatening diabetic retinopathy in patients with type 2 diabetes mellitus: Systematic review, meta-analysis, and prospective validation study
Source: J Glob Health. 2024 Oct 11;14:04192. doi: 10.7189/jogh.14.04192 (PMC11467770; doi:10.7189/jogh.14.04192)
Supplement: Online Supplementary Document [file jogh-14-04192-s001.pdf]

Table S1. Literature search strategy

PubMed

Search number	Query	Results
#1	"diabetic retinopathy"[Title/Abstract] OR "diabetic retinopathies"[Title/Abstract] OR "diabetes mellitus retinopathy"[Title/Abstract] OR "diabetes retinopathy"[Title/Abstract] OR "diabetic retinitis"[Title/Abstract] OR "retinopathia diabetica"[Title/Abstract]	30647
#2	"risk factor"[Title/Abstract] OR "risk factors"[Title/Abstract] OR "population at risk"[Title/Abstract] OR "populations at risk"[Title/Abstract] OR "risk scores"[Title/Abstract] OR "risk score"[Title/Abstract] OR "relative risk"[Title/Abstract] OR "risk factors"[Title/Abstract] OR "prediction factors"[Title/Abstract] OR "prediction factor"[Title/Abstract] OR "logistic models"[Title/Abstract] OR "logistic model"[Title/Abstract] OR "logit models"[Title/Abstract] OR "logit model"[Title/Abstract] OR "logistic regression"[Title/Abstract] OR "logistic regressions"[Title/Abstract] OR "logit regression"[Title/Abstract] OR "cox model"[Title/Abstract] OR "cox multivariate analyses"[Title/Abstract] OR "cox multivariate analysis"[Title/Abstract] OR "cox regression"[Title/Abstract] OR "cox survival analyses"[Title/Abstract] OR "cox survival analysis"[Title/Abstract] OR "cox survival model"[Title/Abstract]	1273863
#3	"VTDR"[Title/Abstract] OR "STDR"[Title/Abstract] OR "Vision-threatening"[Title/Abstract] OR "Sight-threatening"[Title/Abstract] OR "Vision-threatening"[Title/Abstract] OR "Sight-threatening"[Title/Abstract]	5624
#4	#1 AND #2 AND #3	302

Cochrane

Search number	Query	Results
#1	Diabetic Retinopathy	5086
#2	(Diabetic Retinopathy):ti,ab,kw or (Diabetic Retinopathies):ti,ab,kw or (diabetes mellitus retinopathy):ti,ab,kw or (diabetes retinopathy):ti,ab,kw or (diabetic retinitis):ti,ab,kw or (retinopathia diabetica):ti,ab,kw	5137
#3	#1 or #2	5362
#4	(Risk Factor):ti,ab,kw or (Risk factors):ti,ab,kw or (Population at Risk):ti,ab,kw or (Populations at Risk):ti,ab,kw or (Risk Scores):ti,ab,kw or (Risk Score):ti,ab,kw or (relative risk):ti,ab,kw or (risk factors):ti,ab,kw or (prediction factors):ti,ab,kw or (prediction factor):ti,ab,kw or (Logistic Models):ti,ab,kw or (Logistic Model):ti,ab,kw or (Logit Models):ti,ab,kw or (Logit Model):ti,ab,kw or (Logistic Regression):ti,ab,kw or (Logistic Regressions):ti,ab,kw or (logit regression):ti,ab,kw or (Cox model):ti,ab,kw or (Cox multivariate analyses):ti,ab,kw or (Cox multivariate analysis):ti,ab,kw or (Cox regression):ti,ab,kw or (Cox survival analyses):ti,ab,kw or (Cox survival analysis):ti,ab,kw or (Cox survival model):ti,ab,kw	211449
#5	(VTDR):ti,ab,kw or (STDR):ti,ab,kw or (vision-threatening):ti,ab,kw or (sight-threatening):ti,ab,kw or (vision threatening):ti,ab,kw or (sight threatening):ti,ab,kw	479
#6	#3 and #4 and #5	47

Embase

Search number	Query	Results
#1	('diabetic'/exp OR diabetic) AND ('retinopathy'/exp OR retinopathy)	77193
#2	'Diabetic Retinopathies':ab,ti OR 'diabetes mellitus retinopathy':ab,ti OR 'diabetes retinopathy':ab,ti OR 'diabetic retinitis':ab,ti OR 'retinopathia diabetica':ab,ti	574
#3	#1 OR #2	77277
#4	'VTDR':ab,ti OR 'STDR':ab,ti OR 'vision-threatening':ab,ti OR 'sight-threatening':ab,ti OR 'vision threatening':ab,ti OR 'sight threatening':ab,ti	7180
#5	'Risk Factor':ab,ti OR 'Risk Factors':ab,ti OR 'Population at Risk':ab,ti OR 'Populations at Risk':ab,ti OR 'Risk Scores':ab,ti OR 'Risk Score':ab,ti OR 'relative risk':ab,ti OR 'risk factors':ab,ti OR 'prediction factors':ab,ti OR 'prediction factor':ab,ti OR 'Logistic Models':ab,ti OR 'Logistic Model':ab,ti OR 'Logit Models':ab,ti OR 'Logit Model':ab,ti OR 'Logistic Regression':ab,ti OR 'Logistic Regressions':ab,ti OR 'logit regression':ab,ti OR 'Cox model':ab,ti OR 'Cox multivariate analyses ':ab,ti OR 'Cox multivariate analysis':ab,ti OR 'Cox regression':ab,ti OR 'Cox survival analyses':ab,ti OR 'Cox survival analysis':ab,ti OR 'Cox survival model':ab,ti	1845766
#6	#3 and #4 and #5	456

Web of science

Search number	Query	Results
#1	(((((TS=(Diabetic Retinopathy)) OR TS=(Diabetic Retinopathies)) OR TS=(diabetes mellitus retinopathy)) OR TS=(diabetes retinopathy)) OR TS=(diabetic retinitis)) OR TS=(retinopathia diabetica)	48583
#2	(((((TS=(VTDR)) OR TS=(STDR)) OR TS=(Vision-threatening)) OR TS=(Sight-threatening)) OR TS=(Vision threatening)) OR TS=(Sight threatening)	7550
#3	(((((((((((((((((((((TS=(Risk Factor)) OR TS=(Population at Risk)) OR TS=(Populations at Risk)) OR TS=(Risk Scores)) OR TS=(Risk Score)) OR TS=(relative risk)) OR TS=(risk factors)) OR TS=(prediction factors)) OR TS=(prediction factor)) OR TS=(Logistic Models)) OR TS=(Logistic Model)) OR TS=(Logit Models)) OR TS=(Logit Model)) OR TS=(Logistic Regression)) OR TS=(Logistic Regressions)) OR TS=(logit regression)) OR TS=(Cox model)) OR TS=(Cox multivariate analyses)) OR TS=(Cox multivariate analysis)) OR TS=(Cox regression)) OR TS=(Cox survival analyses)) OR TS=(Cox survival analysis)) OR TS=(Cox survival model)	2703672
#4	#1 and #2 and 3	575

Ovid

Search number	Query	Results
#1	(Diabetic Retinopathy OR Diabetic Retinopathies OR diabetes mellitus retinopathy OR diabetes retinopathy OR diabetic retinitis OR retinopathia diabetica).ti,ab,kw.	30620
#2	(Risk Factor OR Risk Factors OR Population at Risk OR Populations at Risk OR Risk	

	Scores OR Risk Score OR relative risk OR risk factors OR prediction factors OR prediction factor OR Logistic Models OR Logistic Model OR Logit Models OR Logit Model OR Logistic Regression OR Logistic Regressions OR logit regression OR Cox model OR Cox multivariate analyses OR Cox multivariate analysis OR Cox regression OR Cox survival analyses OR Cox survival analysis OR Cox survival model).ti,ab,kw.	1273935
#3	(VTDR or STDR or vision-threatening or sight-threatening or vision threatening or sight threatening).ti,ab,kw.	5626
#4	#1 AND #2 AND #3	300

Scopus

Search number	Query	Results
#1	TITLE-ABS-KEY ("Diabetic Retinopathy" OR "Diabetic Retinopathies" OR "diabetes mellitus retinopathy" OR "diabetes retinopathy" OR "diabetic retinitis" OR "retinopathia diabetica")	62260
#2	TITLE-ABS-KEY ("Risk Factor" OR "Population at Risk" OR "Populations at Risk" OR "Risk Scores" OR "Risk Score" OR "relative risk" OR "risk factors" OR "prediction factors" OR "prediction factor" OR "Logistic Models" OR "Logistic Model" OR "Logit Models" OR "Logit Model" OR "Logistic Regression" OR "Logistic Regressions" OR "logit regression" OR "Cox model" OR "Cox multivariate analyses " OR "Cox multivariate analysis" OR "Cox regression" OR "Cox survival analyses" OR "Cox survival analysis" OR "Cox survival model")	2252629
#3	TITLE-ABS-KEY ("VTDR" OR "STDR" OR "vision-threatening" OR "sight-threatening" OR "vision threatening" OR "sight theatening")	5771
#4	#1 AND #2 AND #3	489

ProQuest

Search number	Query	Results
#1	AB,TI("Diabetic Retinopathy" OR "Diabetic Retinopathies" OR "diabetes mellitus retinopathy" OR "diabetes retinopathy" OR "diabetic retinitis" OR "retinopathia diabetica")	53206
#2	AB,TI("VTDR" OR "STDR" OR "Vision-threatening" OR "Sight-threatening" OR "Vision threatening" OR "Sight threatening")	9709
#3	AB,TI("Risk Factor" OR "Population at Risk" OR "Populations at Risk" OR "Risk Scores" OR "Risk Score" OR "relative risk" OR "risk factors" OR "prediction factors" OR "prediction factor" OR "Logistic Models" OR "Logistic Model" OR "Logit Models" OR "Logit Model" OR "Logistic Regression" OR "Logistic Regressions" OR "logit regression" OR "Cox model" OR "Cox multivariate analyses " OR "Cox multivariate analysis" OR "Cox regression" OR "Cox survival analyses" OR "Cox survival analysis" OR "Cox survival model")	2390333
#4	#1 AND #2 AND #3	556

Table S2. Baseline characteristics of the 18 cohort studies included in the systematic review and meta-analysis

Author/Year	Source of cohort (district or country)	Study design and period	Sample Size（male,%）	No. of VTDR initiation	Follow-up (years)	diagnostic criteria of VTDR	Risk factors for VTDR initiation
Manjula D/2022(1)	SIVS1057 (UK)	Prospective cohort 2007-2017	40334 (21676, 53.7%)	1427	3	According to the American Academy of Ophthalmology International Classification, severe non-proliferative diabetic retinopathy, proliferative diabetic retinopathy or diabetic macular oedema.	age, HbA1c, duration of diabetes Age by duration interaction, Gender, Antidiabetic History, History of Background (mild or moderate) diabetic retinopathy
Yen/2021(2)	NHIRD (China)	Retrospective cohort 2000-2015	57886 (30968, 53.5%)	1481	3.69	sight-threatening retinopathy defined as patients with at least two outpatient visits or one admission for retinopathy requiring surgery or receiving laser photocoagulation within 90 days of retinopathy diagnosis, or with visual loss, or receiving anti-vascular endothelial growth factor injection (ranibizumab, bevacizumab, or aflibercept)	Hypertention (**multivariable analysis including sex, age, obesity, smoking status, comorbidities, CCI, DCSI scores, medications, number of oral antidiabetic drugs, and diabetes or hypertension duration.**)

Zhang/2018(3)	Diabetes center of Four ffiliated Hospital of Harbin Medical University (China)	Prospective cohort 2009-2010	738 (376, 50.9%)	61	5	vision-threatening DR was defined as the presence of PDR and/or DME.	fatty acid binding protein 4 (FABP4) **(Adjusted for age and sex, plus established risk factors of DR, including duration of diabetes, HbA1c, SBP, high albuminuria, use of insulin)**
Mahsa/2022(4)	TLGS (Iran)	Prospective cohort 1999-2005.	1169 (494, 42.3%)	187	12.7	severe non-proliferative diabetic retinopathy and proliferative diabetic retinopathy	sex, age, central obesity, BMI, smoking status, educatiom level, blood pressure categories, FPG level categories, glocose-lowering medications
Pedro/2018(5)	DM patients registered in spanish HCA (Spain)	Prospective cohort 2007-2016	15811 (8875, 56.1%)	1451	10	STDR = level 43 or worse as defined by the ETDRS.	age, sex, insulin treatment, arteri hypertension, HbA1c, eGFR, UACR
Ramachandran/ 2020(6)	DEMR system in Chennai, South India (India)	Prospective cohort 2011	17660 (11566, 65.5%)	980	3.9±1.9	STDR was defined by the presence of PDR and/or DME.	serum creatinine, eGFR, albuminuria **(adjusted for age, gender, body mass index, systo blood pressure, fasting blood sugar, glycated hemoglobin, duration of diabetes, cholesterol,**

							**triglyceride and high- density lipoprotein)**
Manjula D /2021(7)	CCGs in East London (UK)	Prospective cohort 2007–2017	58216 (31261, 54.3%)	2757	10	Those who had severe Non-Proliferative DR, Proliferative DR, grading classification for proliferative DR-R3 or grading classification for maculopathy-M1, were identified as people with STDR	ethnic group, duration of diabete age at study entry, gender, townsened score, BMI, HbA1c, SBP, Total Cholesterol, eGFR, history of cardiovascular disease history of antidiabetic drugs hist history of antihypertensives, history of statin
A. Misra/2009(8)	people with diabetes screened by the Central Norfolk DRSS (UK)	Retrospective cohort 1990-2006	20788 (NA)	384	17	STDR [proliferative retinopathy (R3) and treatable maculopathy (M1)]	age, diabetes treatment, years si diabetes diagnosed, blood pressu treatment, months since last screened, each additional screen round period
Elana/2022(9)	a U.S. medical claims database (USA)	Prospective cohort 2002-2019	150252 (77151, 51.3%)	27325	≥1 (1-17)	vision threatening DR defined as a new occurrence of either DME or PDR	age, gender, race, DCSI, Hemoglobin A1c, Hemoglobin A1c Unknown, Use of oral statin **(Covariates controlled for including age, gender, statin us office visits, hypertension diagnosis in addition to drug u**

							**ischemic heart disease, chronic heart disease, dialysis, ischemi stroke, heart failure, peripheral arterial disease, severity of diabetic disease (amputation, diabetic foot, diabetic neuropathy, no diseas charleson comorbidity index))**
Ailsa J/2021(10)	the UK CPRD GOLD database (UK)	Prospective cohort 2010-2011	60094 (33201, 55.3%)	832	3.8±2.0	Sight-threatening DR includes severe NPDR, PDR, and DME	HbA1c, blood pressure, cholesterol, all QOF indicators **(*adjusted for age, sex, ethnicity, index of multiple deprivation, practice region, body mass index, smoking status, alcohol consumption, number of other co-morbid conditions, hospitalisations, duration of diabetes, diabetes complications, number of glucose-lowering therapies, and insulin prescription status)**
Lin/2014(11)	NHIRD () (Taiwan,	Retrospective cohort	21974 (10590, 48.2%)	1221	1.0~1.5	Patients with STDR were identified according to	Insulin treatment **(Adjusted by variables: age, male,**

	China)	2004-2006				ETDRS for clinically significant macular edema and/or severe non-proliferative retinopathy or a code for proliferative retinopathy.	**diabetic-related complication, hypertension, CKD, HbA1c，LDL, antihypertensive agent use and so on)**
Lin/2015(12)	LHID (Taiwan, China)	Retrospective cohort 2000-2011	24562 (12188, 49.6%)	1682	12	STDR includes clinically significant macular edema and/or severe non-proliferative retinopathy according to ETDRS, or clinically significant macular edema and/or proliferative retinopathy.	Antihypertensive Drugs (**Adjusted for variables: age, gender, CVD, hypertension, CKD, insulin, stain use, aspirin use,)**
Li/2018(13)	BDDES (Beijing, China)	Prospective cohort 2009-2012	1438 (575, 40%)	72	5~10	Vision-threatening DR (VTR) was defined as the presence of severe NPDR, PDR, or CSME.	sex, monthly outcome, education, duration of diabetes, age of diabetic onset, SBP, HbA1c, high albuminuria, use of insulin
Li/2022(14)	GDES in China (China)	Prospective cohort 2017-2019	2305 (1317, 57.1%)	98	2	VTDR was defined as the presence of DME or PDR	BMI, WHR (waist to hip ratio), WHtR(waist to height ratio), BAI (Body Adiposity Index)

							**(adjusted for other variables: continuous variables (eg, age, systolic blood pressure, HbA1c, C reaction protein, total cholesterol, triglycerides, low- density cholesterol, high- density cholesterol, creatinine, microalbuminuria, uric acid and axial length) and categorical variables (eg, sex, smoking history, drinking history, education, duration of diabetes and insulin use))**
Huang/2015(15)	The endocrinology department of Zhongnan Hospital (Wuhan, China)	Prospective cohort 2012-2014	324 (180, 55.6%)	41	NA	Vision threatening diabetic retinopathy was defined as the presence of PDR and/or DME.	MBL (Mannose-Binding Lectin), male sex, HbA1c, diabetes durati[illegible] Hs-CRP (High-sensitivity- C-reac[illegible] protein), intensive glucose treatment, hypertension
Han/2022(16)	a community in	Prospective cohort	1370 (595, 43.4%)	11	2	VTDR was defined as the presence of	BMI, WHR (waist to hip ratio)（**adjusted for HbA1c,**

	Guangzhou (Guangzhou, China)	2017-2019				PDR and/or DME	**duration of diabetes, use of insulin, SBP , DBP, total cholesterol, and triglycerides. Bold indicates statistical significance.)**
Han/2021(17)	LECS (Zhaoqing, China)	Retrospective cohort 2008-2010	411 (260, 63.3%)	48	6~8	VTDR, which was defined as pre-proliferative DR or worse, diabetic macular oedema or both.	BMI **(age, systolic blood pressu fasting plasma glucose, total cholesterol and high- density lipoprotein)**
Hu/2021(18)	Shanghai General Hospital (Shanghai, China)	Retrospective cohort 2007-2016	649 (318, 49.0%)	95	9.7	VTDR was defined as the presence of severe non-proliferative or proliferative DR and/or clinically significant macular edema	age, diabetic duration, sub-VTDR, albuminuia, HbA1c, serum magnesium, serum calcium **(adjusted for age, diabetes duration, albuminuria, sub-VTDR, HbA1c, and serum magnesium and corrected serum calcium.)**

Lee/2023(19)	Hong Kong West Diabetes Registry (HKWDR) (Hongkong, China)	Prospective cohort study 2008-2021	4760 (2780, 58.4%)	172	8.8 (7.5-9.7)	STDR represents the presence of severe forms of DR including proliferative DR (PDR) and/or diabetic maculopathy	Men, Age, BMI, Diabetes Duratic Systolic BP, HbA1c, LDL-C, TG eGFR, Presence of A3 albuminur Use of ACEI/ARB, Use of glitazo Use of statin, Use of fibrate, Preexisting NPDR, hsCRPb, AFABPb, PEDFb,
Tsui/2023(20)	Optum's de-identified Clinformatics® Data Mart Database (USA)	Retrospective matched-cohort study (2000-2022)	4158 (2177, 52.4%)	394	1.04 (0.43-2.11)	vision-threatening diabetic retinopathy (VTDR), defined as either diabetic macular edema (DME) or proliferative diabetic retinopathy (PDR)	Use of Erythropoiesis-Stimulating Agents (ESA) **(Covariates Variables of interest included age, gender, race, history of education, income, region of the country, smoking, hypertension, hypercholesterolemia, history of malignancy, insulin use, fenofibrate use, serum creatinine level (mg/dL) and health care usage in the year prior to index)**

Olvera-Barrios/2023(21)	North East London DESP cohort data (UK)	Retrospective cohort study 2012-2021	137591 (73 840 ,54%)	16388	5.4 (2.8–8.2)	grades in order of increasing severity are: no retinopathy (R0), mild non- proliferative diabetic retinopathy (R1), severe non-proliferative diabetic retinopathy (R2), diabetic maculopathy (M1), and proliferative diabetic retinopathy (R3). Sight- threatening diabetic retinopathy (or referable diabetic retinopathy) comprises retinopathy grades greater than or equal to R2	Age, Age category, Sex, Baseline DR grade, Ethnicity, Duration of diabetes, Type of diabetes, Deprivation

Table S3. Newcastle-Ottawa Quality Assessment Scale of the 18 cohort studies

	Selection					Outcome			
Study (First author/Year)	Representative-ness of the exposed cohort	Selection of the non-exposed cohort	Ascertain-ment of exposure	Demonstration that outcome of interest was not present at start of study	**Compara-bility**	Assess-ment of outcome	Was Follow-up Long enough for outcomes to occur	Adequacy of follow up of cohorts	Total scores
Manjula D/2022	★	★	★	★	★★	★	★	★	9
Yen/2021	★	★	★	★	★★	★	★		8
Zhang/2018		★	★	★	★★	★	★	★	8
Mahsa/2022	★	★	★	★	★★	★	★		8
Pedro/2018	★	★	★	★	★★	★	★	★	9
Ramachandran/2020	★	★	★	★	★★	★	★		8
Manjula D /2021	★	★		★	★★	★	★	★	8
A. Misra/2009	★	★	★	★	★★	★	★		8
Elana/2022	★	★	★	★	★★	★	★		8
Ailsa J/2021	★	★	★	★	★★	★	★		8
Lin/2014	★	★	★	★	★★	★	★		8
Lin/2015	★	★	★	★	★★	★	★		8
Li/2018	★	★	★	★	★★	★	★	★	9
Li/2022	★	★	★	★	★★	★	★		8
Huang/2015		★	★	★	★★	★	★		7
Han/2022		★	★	★	★★	★	★	★	8
Han/2021		★	★	★	★★	★	★		7
Hu/2021		★	★	★	★★	★	★	★	8
Lee/2023	★	★	★	★	★★	★	★		8

Tsui/2023	★	★	★	★	★★	★		7
Olvera- Barrios/2023	★	★	★	★	★★	★	★	8

Table S4. 14 predictors included in the systematic review and meta-analysis

Risk factors	First author/Year	Definition of risk factor	RR	95%CI
Age (diabetic onset)	Manjula D/2022	Age (55-64)	0.97	0.95-1
Age (diabetic onset)	Manjula D/2022	Age (65-74)	0.96	0.93-0.98
Age (diabetic onset)	Manjula D/2022	Age (75+)	0.95	0.92-0.97
Age (diabetic onset)	Li/2018	>50	0.153	0.055-0.422
Race	Manjula D/2021(1)	Asian (India)	0.86	0.6-1.21
Race	Manjula D/2021(1)	Asian (Pakistani)	1.36	0.95-1.94
Race	Manjula D/2021(1)	Asian (Bangladeshi)	0.8	0.61-1.05
Race	Manjula D/2021(1)	Black (Caribbean)	1.07	0.98-1.17
Race	Manjula D/2021(1)	Black (African)	1.16	1.07-1.26
Race	Manjula D/2021(1)	Mixed	1.02	0.86-1.21
Race	Manjula D/2021(1)	Other	1.1	1.03-1.19
Race	Manjula D/2021(2)	Asian (India)	1.39	1.18-1.63
Race	Manjula D/2021(2)	Asian (Pakistani)	1.28	1.05-1.55
Race	Manjula D/2021(2)	Asian (Bangladeshi)	1.36	1.19-1.55
Race	Manjula D/2021(2)	Black (Caribbean)	1.22	1.04-1.43
Race	Manjula D/2021(2)	Black (African)	1.1	0.92-1.33
Race	Manjula D/2021(2)	Mixed	1.17	0.85-1.61
Race	Manjula D/2021(2)	Other	1.25	1.07-1.47
Race	Elana/2022	Other	0.95	0.88-1.03
Race	Elana/2022	Mixed (Hispannic)	0.96	0.93-1
Race	Elana/2022	Asian (Asian)	0.81	0.76-0.87
Race	Elana/2022	Black (Black)	0.91	0.88-0.95
Race	Olvera- Barrios/2023	Asian (South Asian)	1.36	1.31-1.42
Race	Olvera- Barrios/2023	Black	1.57	1.50-1.64

Race	Olvera- Barrios/2023	Asian (Any other Asian)	1.25	1.16-1.34
Race	Olvera- Barrios/2023	Other	1.29	1.18-1.42
Race	Olvera- Barrios/2023	Mixed	1.39	1.20-1.60
Race	Olvera- Barrios/2023	Asian (Chinese)	0.98	0.79-1.21
Gender	Manjula D/2022	Male	1.124	1.01-1.25
Gender	Mahsa/2022	Male	0.79	0.54-1.16
Gender	Pedro/2018	Male	0.644	0.531-0.781
Gender	Elana/2022	Male	0.93	0.9-0.95
Gender	Li/2018	Male	1.28	0.59-2.76
Gender	Huang/2015	Male	1.08	1.02-1.35
Gender	Lee/2023	Male	1.03	0.72-1.47
Gerder	Olvera- Barrios/2023	Male	1.04	1.01-1.07
Stain use	Manjula D/2021	stain use	1.2	0.99-1.46
Stain use	Manjula D/2021	stain use	0.96	0.85-1.08
Stain use	Elana/2022	stain use	1.01	0.99-1.04
Stain use	Lee/2023	stain use	0.61	0.44-0.85
Antihypertensive Medication	Manjula D/2021 (1)	Antihypertensive Medication	0.77	0.63-0.93
Antihypertensive Medication	Manjula D/2021 (2)	Antihypertensive Medication	1	0.89-1.12
Antihypertensive Medication	A. Misra/2009	Antihypertensive Medication	0.71	0.57-0.88
WHR	Li/2022	WHR Quarter 4	1.98	0.34-11.61
WHR	Han/2022	WHR Tertile 3	3.36	0.55-20.36
Albuminuria	Ramachandran/2019	Macroalbuminuria =30-300 µg/mg	1.34	1.2-1.5
Albuminuria	Ramachandran/2020	Macroalbuminuria =>300 µg/mg	2	1.57-2.55
Albuminuria	Hu/2021	Albuminuria (UACR>=30mg/g)	1.58	1.05-2.4
Albuminuria	Lee/2023	Presence of A3 (300mg/g) albuminuria, %	1.57	1.07-2.31
Total Cholesterol	Manjula D/2021	Total Cholesterol =>6.2 mmol/L	0.94	0.75-1.19

Total Cholesterol	Manjula D/2021	Total Cholesterol =>6.2 mmol/L	1.04	0.88-1.24
Total Cholesterol	Ailsa J/2021	Total Cholesterol> 5 mmol/L	1.22	1.01-1.493
eGFR	Pedro/2018	eGFR < 60 ml/min/1.73m²	1.097	0.899-1.979
eGFR	Ramachandran/2020	eGFR 46-60 ml/min/1.73m²	1.68	1.22-2.31
eGFR	Ramachandran/2020	eGFR 30-45 ml/min/1.73m²	1.85	1.14-2.99
eGFR	Ramachandran/2020	eGFR <30 ml/min/1.73m²	4.85	2.87-8.21
eGFR	Manjula D/2021(1)	eGFR < 60 ml/min/1.73m²	1.32	0.97-1.79
eGFR	Manjula D/2021(2)	eGFR < 60 ml/min/1.73m²	1.35	1.19-1.54
eGFR	Lee/2023	eGFR 60-89 ml/min/1.73m²	1.11	0.74-1.67
eGFR	Lee/2023	eGFR 30-59 ml/min/1.73m²	1.03	0.57-1.86
eGFR	Lee/2023	eGFR <30 ml/min/1.73m²	0.73	0.26-2.04
BMI	Mahsa/2022	overweight (25-30kg/m²)	0.6	0.39-0.92
BMI	Mahsa/2022	obese (>30kg/m²)	0.48	0.27-0.83
BMI	Manjula D/2021(1)	overweight (25-30kg/m²)	0.47	0.27-0.82
BMI	Manjula D/2021(1)	obese (>30kg/m²)	0.32	0.18-0.57
BMI	Manjula D/2021(2)	overweight (25-30kg/m²)	0.89	0.63-1.27
BMI	Manjula D/2021(2)	obese (>30kg/m²)	0.76	0.53-1.08
BMI	Li/2022	overweight (23-25kg/m²)	1.03	0.46-2.32
BMI	Li/2022	obese (>25kg/m²)	0.37	0.16-0.87
BMI	Han/2022	overweight (23-27.5kg/m²)	0.18	0.02-1.57
BMI	Han/2022	obese (>27.5kg/m²)	0.26	0.05-1.39
BMI	Han/2021	overweight (25-30kg/m²)	0.79	0.39-1.62
BMI	Han/2021	obese (>30kg/m²)	1.1	0.28-4.28
BMI	Lee/2023	BMI (kg/m²)	0.97	0.93-1.02
Hypertension	Yen/2021	Hypertension (>=140mmHg)	2.07	1.85-2.3

Hypertension	Mahsa/2022	Hypertension (Newly diagnosed hypertensive)	1.96	1.06-3.65
Hypertension	Mahsa/2022	Hypertension (Controlled treated hypertensive)	1.14	0.63-2.08
Hypertension	Mahsa/2022	Hypertension (Uncontrolled treated hypertensive)	1.42	0.87-2.31
Hypertension	Pedro/2018	Hypertension (>=140mmHg)	2.128	1.726-2.623
Hypertension	Manjula D/2021(1)	Hypertension (>=140mmHg)	1.88	1.45-2.44
Hypertension	Manjula D/2021(2)	Hypertension (>=140mmHg)	1.8	1.57-2.05
Hypertension	Ailsa J/2021	Hypertension (>=140mmHg)	1.282	1.099-1.493
Hypertension	Li/2018	Hypertension (>=140mmHg)	1.99	1.03-3.85
Hypertention	Huang/2015	Hypertention (>=140mmHg)	1.58	1.28-2.3
Hypertention	Lee/2023	Systolic BP, mmHg	1.01	1.001-1.02
HbA1c	Manjula D/2022	HbA1c(abnormal)60-69mmol/L/7.6-8.5%	1.69	1.39-2.05
HbA1c	Manjula D/2022	HbA1c(abnormal)70-79mmol/L/8.5-9.4%	1.82	1.47-2.25
HbA1c	Manjula D/2022	HbA1c(abnormal)>80mmol/L />9.4%	2.88	2.39-3.46
HbA1c	Ailsa J/2021	HbA1c (abnormal)>59 mmol/mol/7.5%	1.351	1.149-1.613
HbA1c	Li/2018	HbA1c (abnormal)>59 mmol/mol/7.5%	0.87	0.44-1.7
HbA1c	Pedro/2018	Increment by 1% (11mmol/L)	3.23	2.378-4.387
HbA1c	Elana/2022	Increment by 1% (11mmol/L)	1.04	1.03-1.05
HbA1c	Huang/2015	Increment by 1% (11mmol/L)	1.08	1.03-1.16
HbA1c	Hu/2021	Increment by 1% (11mmol/L)	1.24	1.11-1.39
HbA1c	Lee/2023	Increment by 1% (11mmol/L)	1.39	1.27-1.52
Duration of diabetes	A. Misra/2009	10~20years	2.68	2.03-3.53
Duration of diabetes	A. Misra/2009	>20years	3.79	2.71-5.29
Duration of diabetes	Li/2018	10~15years	2.32	0.66-8.14
Duration of diabetes	Li/2018	>15years	2.28	0.62-8.43

Duration of diabetes	Huang/2015	diabetes duration (per 1 y increase)	1.12	1.04-1.25
Duration of diabetes	Hu/2021	diabetes duration (per 1 y increase)	1.06	1.02-1.1
Duration of diabetes	Manjula D/2022	diabetes duration (per 1 y increase)	1.09	1.06-1.11
Duration of diabetes	Lee/2023	Diabetes duration 5-9 y	1.32	0.64-2.72
Duration of diabetes	Lee/2023	Diabetes duration ≥10 y	3.01	1.63-5.58
Duration of diabetes	Olvera- Barrios/2023	Duration of diabetes (per 5- year increase)	1.14	1.13-1.15
Diabetes treatment	Manjula D/2022	Diabetes treatment (one drug)	1.35	1.05-1.73
Diabetes treatment	Manjula D/2022	Diabetes treatment (two drugs)	2.42	1.91-3.07
Diabetes treatment	Manjula D/2022	Diabetes treatment (insulin)	3.43	2.66-4.42
Diabetes treatment	Pedro/2018	Diabetes treatment (insulin)	1.313	1.125-1.535
Diabetes treatment	A. Misra/2009	Diabetes treatment (Oral drugs)	1.31	0.96-1.8
Diabetes treatment	A. Misra/2009	Diabetes treatment (Insulin)	2.12	1.43-3.14
Diabetes treatment	Li/2018	Diabetes treatment (Insulin)	10.04	4.42-22.81

Table S5. Pooled RRs of the 14 predictors

Factors	No. of studies	Pooled RR (95%CI)	I2
Age of diabetes onset			
<50 (Ref)			
>=50	4	**0.958(0.922,0.995) ***	78.10%
EthnicGroup			
White (Ref)			
Asian	10	1.128(0.958,1.327)	95.3%
Black	6	1.156(0.914,1.461)	98.5%
Mixed	4	1.114(0.911,1.361)	88.1%
Other	4	1.132(0.984,1.303)	89.0%
Gender			
Female (Ref)			
Male	8	0.970(0.887,1.060)	58.83%
BMI			
Normal (Ref)			
Overweight	6	**0.713(0.572,0.889) ***	25.60%
Obese	6	**0.551(0.431,0.706) ***	46.80%
Duration of diabetes			
<10years (Ref)			
10years~	5	**3.035(2.493,3.694) ***	0.00%
Duration of diabetes			
Per 1 y increase	3	**1.083(1.063,1.104) ***	2.60%
HbA1c			
Normal (Ref)			
High	5	**1.703(1.237,2.343) ***	90.20%
HbA1c			
Per 1% increase	5	**1.328(1.139,1.548) ***	96.10%
Per 1% increase (sensitivity analysis)	4	**1.171(1.041,1.317) ***	94.00%
Hypertension			
No (Ref)			
Yes	10	**1.726(1.492,1.998) ***	71.70%
Yes (sensitivity analysis)	9	**1.919(1.789,2.059) ***	18.30%
Total Cholesterol			
Normal (Ref)			
Hypercholesterolemia	3	1.070(0.957,1.198)	34.40%
eGFR(mL/min/1.73m2)			
eGFR=>60 (Ref)			
eGFR<60	8	**1.525(1.171,1.987) ***	80.40%
eGFR<60 (sensitivity analysis)	7	**1.358(1.226,1.505) ***	10.3%
Albuminuria			
NO (Ref)			
YES	4	**1.585(1.265,1.985) ***	66.70%
WHR			

Q1 (Ref)			
Q3~Q4	2	2.564(0.726,9.060)	0.00%
Statin use			
No (Ref)			
Yes	4	0.973(0.850,1.114)	76.40%
Antihypertension			
No (Ref)			
Yes	3	0.829(0.662,1.040)	80.50%
Diabetic treatment			
No (Ref)			
Oral drugs	3	**1.63(1.08,2.47) ***	86.10%
Insulin	4	**2.88(1.47,5.64) ***	94.80%

Table S6. Predictors with the number of involved studies, sample size, pooled RRs (95%CI), β-coefficients, and risk scores included in the VTDR risk prediction model

Risk factor for VTDR	No. of studies	Sample size	Pooled RR	95%CI	β-coefficient	Scores
**Age of diabetes onset**						
>=50y (REF)						
<50y	4	41772	1.044	1.005-1.085	0.043	0.4
**Duration of diabetes**						
Per 1 y increase	3	41307	1.083	1.063-1.104	0.080	0.8
**HbA1c**						
≤7% (Ref)						
Per 1% increase	4	167036	1.171	1.041-1.317	0.158	1.6
**Hypertention**						
No(Ref)						
Yes	9	194938	1.919	1.789-2.059	0.652	6.5
**eGFR（ml/min/1.73m^2）**						
eGFR=>60(Ref)						
eGFR<60	7	91687	1.358	1.226-1.505	0.306	3.1
**High albuminuria**						
No(Ref)						
Yes	4	18309	1.585	1.265-1.985	0.461	4.6
**Diabetic treatment**						
NO(Ref)						
Oral drugs	3	61122	1.63	1.08-2.47	0.489	4.9
Insulin	4	78371	2.88	1.47-5.64	1.058	10.6

Table S7 Baseline characteristic of the validation cohorts

Variables	Validation cohort 1	Validation cohort 2	Total	Development of VTDR		P value
				No	Yes	
**N**	291	264	555	505(90.1%)	50(9.9%)	
**Follow-up (months)**	76(63,93)	39(37,47)	52(39,77)	51(39,75.5)	59(41,98)	0.081
**Age of diabetes onset (year)**	53.5±11.2	47.7±11.2	50.8±11.5	51.2±11.5	46.7±11.0	**0.009***
**Gender(n,%)**						**0.03***
Male	124(42.6%)	139(52.7%)	263(47.4%)	232(45.9%)	31(62.0%)	
Female	167(57.4%)	125(47.3%)	292(52.6%)	273(54.1%)	19(38.0%)	
**Duration of diabetes(months)**	72(24,120)	61(11,124)	71.5(22,123)	63.5(18.3,120)	114(48,150)	**0.001***
**HbA1c(%)**	6.7(6.2,7.8)	7.35(6.3,9.85)	7.6(6.5,9.8)	7.4(6.5,9.5)	10.7(8.1,11.9)	**0.000***
**Hypertension (n,%)**	175(60.1%)	94(35.6%)	269(48.5%)	237(46.9%)	32(64.0%)	**0.021***
**eGFR (<60,n,%)**	69(23.7%)	25(9.5%)	94(16.9%)	80(15.8%)	14(28.0%)	**0.029***
**High albuminuria (n,%)**	46(15.8%)	65(14.6%)	111(20.0%)	84(16.6%)	27(54.0%)	**0.000***
**Insulin use**	154(52.9%)	91(35.5%)	245(44.1%)	200(39.6%)	45(90.0%)	**0.000***
**Oral diabetic medication**	97(33.3%)	249(94.3%)	346(62.3%)	323(64.0%)	23(46.0%)	**0.012***

The baseline characteristics of patients in the validation cohorts included follow-up time, age of diabetes onset, gender, duration of diabetes, glycosylated hemoglobin (HbA1c, %), estimated glomerular filtration rate (eGFR, ml/min/1.73m²), presence of high albuminuria (UACR>30mg/g), use of insulin, and oral hypoglycemic medications. *Patients in the validation cohorts who developed VTDR were statistically different (*P*<0.05) from those who did not develop VTDR.

Table S8-a. Performance of VTDR risk prediction model for 3 years at different cut-off value

Cut-off value	Sensitivity	Specificity
≥0.07	100.00	0.00
**>23.76**	**100.00**	**64.00**
>23.8	80.00	64.00
>36.12	80.00	92.18
>36.22	60.00	92.18
>44.34	60.00	98.00
>44.82	40.00	98.00
>46.12	40.00	98.36
>46.2	20.00	98.36
>49.42	20.00	99.64
>50.12	0.00	99.64
>55.1	0.00	100.00

Table S8-b. Performance of VTDR risk prediction model for 4 years at different cut-off value

Cut-off value	Sensitivity	Specificity
0.07	100.00	0.00
>8.26	100.00	13.17
>8.3	94.44	13.17
**>22.98**	**94.44**	**61.98**
>23.04	83.33	61.98
>23.68	83.33	64.07
>23.8	77.78	64.07
>25.58	77.78	69.16
>25.74	72.22	69.16
>28.12	72.22	77.84
>28.2	66.67	77.84
>30.06	66.67	83.23
>30.28	61.11	83.23
>30.3	61.11	83.53
>30.54	55.56	83.53
>34.14	55.56	90.12
>34.3	50.00	90.12
>34.76	50.00	90.72
>34.8	44.44	90.72

>36.12	44.44	92.22
>36.22	38.89	92.22
>38.52	38.89	95.21
>38.64	33.33	95.21
>39.88	33.33	96.11
>40.16	27.78	96.11
>40.76	27.78	97.01
>40.88	22.22	97.01
>41.8	22.22	97.60
>41.9	16.67	97.90
>44.34	16.67	99.10
>46.2	5.56	99.10
>48.42	5.56	99.70
>50.12	0.00	99.70
>54.8	0.00	100.00

Table S8-c. Performance of VTDR risk prediction model for 5 years at different cut-off value

Cut-off value	Sensitivity	Specificity
≥0.07	100.00	0.00
>8.26	100.00	14.75
>8.3	96.30	14.75
**>22.98**	**96.30**	**62.70**
>23.04	88.89	62.70
>23.68	88.89	65.57
>23.8	85.19	65.57
>25.58	85.19	72.13
>25.74	81.48	72.13
>26.14	81.48	72.95
>26.3	77.78	73.36
>26.6	77.78	75.41
>26.64	74.07	75.41
>27.82	74.07	79.51
>28.2	66.67	79.51
>30.06	66.67	85.66
>30.28	62.96	85.66
>30.3	62.96	86.07
>30.54	59.26	86.07
>30.78	59.26	88.11

>30.88	55.56	88.11
>31.58	55.56	90.16
>32.08	51.85	90.16
>32.44	51.85	90.98
>32.5	48.15	90.98
>33.38	48.15	91.80
>34.3	44.44	91.80
>34.76	44.44	92.62
>34.8	40.74	92.62
>35.34	40.74	93.85
>36.22	37.04	93.85
>38.52	37.04	96.72
>40.16	22.22	96.72
>40.76	22.22	97.54
>40.88	18.52	97.54
>41.9	14.81	97.95
>42.42	14.81	98.77
>43.52	11.11	98.77
>44.34	11.11	99.18
>46.2	3.70	99.18
>48.42	3.70	100.00
>50.12	0.00	100.00

Table S8-d. Performance of VTDR risk prediction model for 6 years at different cut-off value

Cut-off value	Sensitivity	Specificity
≥0.07	100.00	0.00
>8.26	100.00	16.27
>8.3	96.55	16.27
**>22.98**	**96.55**	**62.65**
>23.04	89.66	62.65
>23.61	89.66	65.66
>23.8	86.21	65.66
>25.22	86.21	68.67
>25.74	82.76	68.67
>26.14	82.76	69.88
>26.3	79.31	70.48
>26.6	79.31	72.89
>26.64	75.86	72.89

>27.82	75.86	77.71
>28.25	65.52	77.71
>30.06	65.52	84.34
>30.28	62.07	84.34
>30.3	62.07	84.94
>30.54	58.62	84.94
>30.78	58.62	87.95
>30.88	55.17	87.95
>31.58	55.17	89.76
>32.08	51.72	89.76
>32.44	51.72	90.96
>32.5	48.28	90.96
>33.38	48.28	91.57
>34.3	44.83	91.57
>34.76	44.83	92.77
>34.8	41.38	92.77
>35.34	41.38	93.98
>36.22	37.93	93.98
>38.52	37.93	96.99
>40.16	24.14	96.99
>40.76	24.14	97.59
>40.88	20.69	97.59
>41.9	17.24	98.19
>42.26	17.24	98.80
>43.52	10.34	98.80
>44.34	10.34	99.40
>46.2	3.45	99.40
>48.42	3.45	100.00
>50.12	0.00	100.00

Table S8-e. Performance of VTDR risk prediction model for 7 years at different cut-off value

Cut-off value	Sensitivity	Specificity
≥0.07	100.00	0.00
>8.26	100.00	18.02
>8.3	96.87	18.02
>19.21	96.87	51.35
>19.24	93.75	51.35
**>22.72**	**93.75**	**63.96**

>23.04	87.50	63.96
>23.61	87.50	66.67
>23.8	84.37	66.67
>25.22	84.37	70.27
>25.74	81.25	70.27
>25.82	81.25	71.17
>26.3	78.12	72.07
>26.38	78.12	72.97
>26.64	75.00	72.97
>27.82	75.00	78.38
>28.25	65.62	78.38
>30.06	65.62	86.49
>30.54	59.38	86.49
>30.7	59.38	88.29
>30.72	56.25	88.29
>30.78	56.25	89.19
>31.22	50.00	89.19
>31.32	50.00	90.09
>32.08	46.88	90.09
>32.44	46.88	90.99
>32.5	43.75	90.99
>33.38	43.75	91.89
>34.3	40.63	91.89
>34.68	40.63	92.79
>36.22	34.38	92.79
>38.52	34.38	96.40
>40.16	21.87	96.40
>40.76	21.87	97.30
>41.9	15.63	97.30
>42.26	15.63	98.20
>43.52	9.38	98.20
>44.34	9.38	99.10
>46.2	3.12	99.10
>48.42	3.12	100.00
>50.12	0.00	100.00

Table S8-f. Performance of VTDR risk prediction model for 8 years at different cut-off value

Cut-off value	Sensitivity	Specificity

≥1.6	100.00	0.00
>8.26	100.00	22.22
>8.3	97.30	22.22
>19.2	97.30	47.62
>19.24	94.59	47.62
>21.9	94.59	58.73
>22.2	91.89	58.73
**>22.72**	**91.89**	**61.90**
>23.04	86.49	61.90
>23.61	86.49	65.08
>23.8	83.78	65.08
>24.193	83.78	68.25
>25.74	81.08	68.25
>25.82	81.08	69.84
>26.64	75.68	69.84
>27.66	75.68	77.78
>28.25	67.57	77.78
>28.8	67.57	80.95
>28.88	64.86	80.95
>29.42	64.86	84.13
>29.68	62.16	84.13
>30	62.16	85.71
>30.54	56.76	85.71
>30.62	56.76	87.30
>31.22	48.65	87.30
>31.32	48.65	88.89
>32.08	45.95	88.89
>32.44	45.95	90.48
>34.3	40.54	90.48
>34.68	40.54	92.06
>36.22	35.14	92.06
>38.52	35.14	96.83
>41.9	16.22	96.83
>42.26	16.22	98.41
>43.52	10.81	98.41
>44.34	10.81	100.00
>50.12	0.00	100.00

Table S8-g. Performance of VTDR risk prediction model for 9 years at different

cut-off value

Cut-off value	Sensitivity	Specificity
≥1.6	100.00	0.00
>8.26	100.00	29.03
>8.3	97.78	29.03
>17.5	97.78	54.84
>19.24	93.33	54.84
**>21.9**	**93.33**	**70.97**
>23.8	82.22	70.97
>23.9	82.22	74.19
>27.44	71.11	74.19
>27.66	71.11	80.65
>28.88	60.00	80.65
>29.42	60.00	87.10
>29.68	57.78	87.10
>30	57.78	90.32
>30.54	53.33	90.32
>30.62	53.33	93.55
>31.22	46.67	93.55
>31.32	46.67	96.77
>36.22	35.56	96.77
>38.26	35.56	100.00
>50.12	0.00	100.00

Table S8-h. Performance of VTDR risk prediction model for 10 years at different cut-off value

Cut-off value	Sensitivity	Specificity
≥4	100.00	0.00
>7.43	100.00	25.00
>8.3	97.87	25.00
>15.68	97.87	50.00
>19.24	93.62	50.00
**>21.9**	**93.62**	**81.25**
>27.44	72.34	81.25
>27.6	72.34	87.50
>30.54	51.06	87.50
>30.62	51.06	93.75
>36.22	34.04	93.75
>38.26	34.04	100.00

>50.12	0.00	100.00

Severity Level	Findings after pupil dilation	VTDR
**DR scale**		
**No DR**	No abnormalitics	
**Mild NPDR**	Microaneurysms only	
**Moderate NPDR**	More than just microaneurysms but less than Severe NPDR	
**Severe NPDR**	Any of the following:<br>● >20 intraretinal hemorrhages in each of 4 quadrants;<br>● Definite venous beading in 2+ quadrants;<br>● Prominent IRMA in 1+ quadrant;<br>And no signs of proliferative retinopathy	
**PDR**	One or more of the following:<br>● Neovascularization;<br>● Vitreous/preretinal hemorrhage	
**DME scale**		
**No DME**	No apparent retinal thickening or hard exudates in posterior pole	
**DME**	Some retinal thickening or hard exudates in posterior pole:<br>● Mild: some retinal thickening or hard exudates in posterior pole but distant from the center of the macular;<br>● Moderate: retinal thickening or hard exudates approaching the center of macula but not involving the center.<br>● Severe: retinal thickening or hard exudates involving the center of the macular.	

Figure S1 International Clinical Severity Scale of Diabetic Retinopathy and Diabetic Macular Oedema. DME, diabetic macular oedema; DR, diabetic retinopathy; IRMA, intraretinal microvascular abnormalities; NPDR, non- proliferative diabetic retinopathy; PDR, proliferative diabetic retinopathy; VTDR, vision- threatening DR.

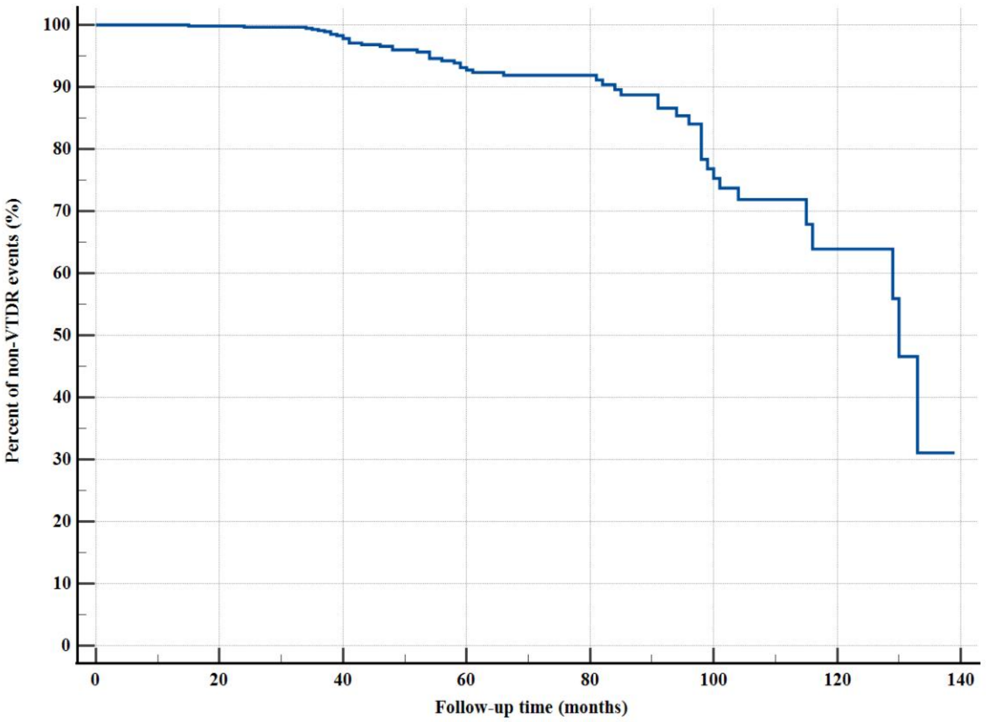

Figure S2 Survival curve of the VTDR endpoint of the validation cohorts

References

1. Nugawela MD, Gurudas S, Prevost AT, Mathur R, Robson J, Sathish T, et al. Development and validation of predictive risk models for sight threatening diabetic retinopathy in patients with type 2 diabetes to be applied as triage tools in resource limited settings. EClinicalMedicine. 2022;51:101578.
2. Yen FS, Wei JCC, Shih YH, Hsu CC, Hwu CM. The Risk of Nephropathy, Retinopathy, and Leg Amputation in Patients With Diabetes and Hypertension: A Nationwide, Population-Based Retrospective Cohort Study. Frontiers in Endocrinology. 2021;12.
3. Xian-zhao Z, Wen-Jun T, Wang H, Zhao Q, Liu Q, Sun L, et al. Circulating Serum Fatty Acid-Binding Protein 4 Levels Predict the Development of Diabetic Retinopathy in Type 2 Diabetic Patients. American Journal of Ophthalmology. 2018;187:71-9.
4. Sardarinia M, Asgari S, Arani RH, Eskandari F, Azizi F, Khalili D, et al. Incidence and risk factors of severe non-proliferative/proliferative diabetic retinopathy: More than a decade follow up in the Tehran Lipids and Glucose Study. Journal of Diabetes Investigation. 2022;13(2):317-27.
5. Romero-Aroca P, Baget-Bernaldiz M, Navarro-Gil R, Moreno-Ribas A, Valls-Mateu A, Sagarra-Alamo R, et al. Glomerular Filtration Rate and/or Ratio of Urine Albumin to Creatinine as Markers for Diabetic Retinopathy: A Ten-Year Follow-Up Study. J Diabetes Res. 2018;2018:5637130.
6. Rajalakshmi R, Rani CSS, Venkatesan U, Unnikrishnan R, Anjana RM, Rani SJ,

et al. Correlation between markers of renal function and sight-threatening diabetic retinopathy in type 2 diabetes: a longitudinal study in an Indian clinic population. Bmj Open Diabetes Research & Care. 2020;8(1).

7. Nugawela MD, Gurudas S, Prevost AT, Mathur R, Robson J, Hanif W, et al. Ethnic Disparities in the Development of Sight-Threatening Diabetic Retinopathy in a UK Multi-Ethnic Population with Diabetes: An Observational Cohort Study. Journal of Personalized Medicine. 2021;11(8):28.

8. Misra A, Bachmann MO, Greenwood RH, Jenkins C, Shaw A, Barakat O, et al. Trends in yield and effects of screening intervals during 17 years of a large UK community-based diabetic retinopathy screening programme. Diabetic medicine : a journal of the British Diabetic Association. 2009;26(10):1040-7.

9. Meer E, Bavinger JC, Yu Y, Hua P, McGeehan B, VanderBeek BL. Statin use and the risk of progression to vision threatening diabetic retinopathy. Pharmacoepidemiology and drug safety. 2022;31(6):652-60.

10. McKay AJ, Gunn LH, Sathish T, Vamos E, Nugawela M, Majeed A, et al. Associations between attainment of incentivised primary care indicators and incident diabetic retinopathy in England: a population-based historical cohort study. BMC Medicine. 2021;19(1).

11. Lin JC, Shau WY, Lai MS. Long-acting insulin analogues and diabetic retinopathy: a retrospective cohort study. Clin Ther. 2014;36(9):1255-68.

12. Lin JC, Lai MS. Antihypertensive Drugs and Diabetic Retinopathy in Patients with Type 2 Diabetes. Ophthalmologica. 2016;235(2):87-96.

13. Li YY, Yang XF, Gu H, Liu XP, Snellingen T, Liu NP. The Beijing Desheng Diabetic Eye Study: rationale, design, methodology and baseline data. Int J Ophthalmol. 2018;11(1):108-16.

14. Li W, Gong X, Wang W, Xiong K, Meng J, Li Y, et al. Association of different kinds of obesity with diabetic retinopathy in patients with type 2 diabetes. BMJ open. 2022;12(5):e056332.

15. Huang Q, Shang G, Deng H, Liu J, Mei Y, Xu Y. High Mannose-Binding Lectin Serum Levels Are Associated with Diabetic Retinopathy in Chinese Patients with Type 2 Diabetes. PloS one. 2015;10(7):e0130665.

16. Han XY, Wu HM, Li YJ, Yuan M, Gong X, Guo X, et al. Differential Effect of Generalized and Abdominal Obesity on the Development and Progression of Diabetic Retinopathy in Chinese Adults With Type 2 Diabetes. Frontiers in Medicine. 2022;9.

17. Han X, Jiang Y, Niu Y, Zhu Y, Huang W, He M. Differential associations between body mass index with diabetes and vision-threatening diabetic retinopathy in an adult Chinese population. British Journal of Ophthalmology. 2022;106(6):852-6.

18. Hu Y, Zhou C, Shi Y, She X, Zhao S, Gu C, et al. A Higher Serum Calcium Level is an Independent Risk Factor for Vision-Threatening Diabetic Retinopathy in Patients with Type 2 Diabetes: Cross-Sectional and Longitudinal Analyses. Endocrine practice : official journal of the American College of Endocrinology and the American Association of Clinical Endocrinologists. 2021;27(8):826-33.

19. Lee CH, Lui DTW, Cheung CYY, Fong CHY, Yuen MMA, Woo YC, et al. Circulating AFABP, FGF21, and PEDF Levels as Prognostic Biomarkers of Sight-threatening Diabetic Retinopathy. Journal of Clinical Endocrinology & Metabolism. 2023;108(9):E799-E806.

20. Tsui JC, Willett K, Cohen JB, Yu YX, VanderBeek BL. Erythropoiesis-Stimulating Agents and the Risk of Vision-Threatening Diabetic Retinopathy. Ophthalmic Epidemiology. 2023.

21. Olvera-Barrios A, Owen CG, Anderson J, Warwick AN, Chambers R, Bolter L, et al. Ethnic disparities in progression rates for sight-threatening diabetic retinopathy in diabetic eye screening: a population-based retrospective cohort study. BMJ open diabetes research & care. 2023;11(6).
